# Supplementary material for: Direct, indirect, and vicarious nature experiences collectively predict preadolescents’ self-reported nature connectedness and conservation behaviors
Source: PeerJ. 2023 Jun 21;11:e15542. doi: 10.7717/peerj.15542 (PMC10290449; doi:10.7717/peerj.15542)
Supplement: Supplemental Information 3 — The original questionnaire used in the survey is in Chinese. [file peerj-11-15542-s003.docx]

**Supplementary materials: Questionnaire** **used in the survey (In Chinese).**

Hi!

This is a survey on the "Relationship between human and nature", I am very happy that you can participate. According to your true feelings, please draw a “√” in □. You may only select one answer from the five options.

**Your name**： **Your gender：**

**Your age ：** **Your residence：**

|  | Using “🗸” Choose your opinion on the following sentences. | Strongly disagree | Disagree | Not sure | Agree | Strongly agree |
| --- | --- | --- | --- | --- | --- | --- |
| 1 | Humans are part of the natural world. |  |  |  |  |  |
| 2 | People cannot live without plants and animals. |  |  |  |  |  |
| 3 | Nature is the common home of animals, plants, and humans. |  |  |  |  |  |
| 4 | I feel sad when wild animals are hurt. |  |  |  |  |  |
| 5 | I like animals to be free, not caged. |  |  |  |  |  |
| 6 | It makes me sad to see deforestation. |  |  |  |  |  |
| 7 | Being outdoors makes me happy. |  |  |  |  |  |
| 8 | I like to hear different sounds in nature. |  |  |  |  |  |
| 9 | I like to see wild flowers in nature. |  |  |  |  |  |
| 10 | When I feel sad, I like to go outside and enjoy nature. |  |  |  |  |  |
| 11 | Collecting rocks and shells is fun. |  |  |  |  |  |
| 12 | I enjoy touching animals and plants. |  |  |  |  |  |
| 13 | Taking care of animals is important to me. |  |  |  |  |  |

**[Single Choice] Recall your performance in the past year; please tick "🗸" under the option that best matches your real situation.**

|  |  | Never | Rarely | Sometimes | Often | Always |
| --- | --- | --- | --- | --- | --- | --- |
| 1 | I carry out activities to protect the environment. |  |  |  |  |  |
| 2 | To save water, I use less water when I take a shower or bath. |  |  |  |  |  |
| 3 | I talk to my teachers and peers at school about the importance of doing things to protect the environment. |  |  |  |  |  |
| 4 | I help separate (trash) and recycle at home. |  |  |  |  |  |
| 5 | I switch off electrical appliances when I am not using them to save energy. |  |  |  |  |  |
| 6 | I pick up litter to help nature have a better home. |  |  |  |  |  |
| 7 | I put food out to feed garden birds. |  |  |  |  |  |
| 8 | I make homes for nature at school or in the garden. |  |  |  |  |  |
| 9 | I put insects stuck inside safely outside. |  |  |  |  |  |
| 10 | I grow flowers and plants that birds and insects will like. |  |  |  |  |  |
| 11 | I take part in events to help nature (e.g. bird watching). |  |  |  |  |  |
| 12 | I am a member of a wildlife or nature group at school. |  |  |  |  |  |
| 13 | I am a member of a wildlife or nature group outside of school. |  |  |  |  |  |

**[Single Choice] How often have you participated in the following activities in the past year? Please tick "🗸" under the option that best suits your personal situation**

|  |  | Never | Rarely | Sometimes | Often | Always |
| --- | --- | --- | --- | --- | --- | --- |
| 1 | Catch fish and tadpoles |  |  |  |  |  |
| 2 | Climb tree |  |  |  |  |  |
| 3 | Picking fruit to eat |  |  |  |  |  |
| 4 | Play mud |  |  |  |  |  |
| 5 | Planting flowers and trees |  |  |  |  |  |
| 6 | Mountaineer |  |  |  |  |  |
| 7 | Fly a kite |  |  |  |  |  |
| 8 | Collect natural things |  |  |  |  |  |
| 9 | Observe insects |  |  |  |  |  |
| 10 | Visit the Zoo |  |  |  |  |  |
| 11 | Visit the Botanical Garden |  |  |  |  |  |
| 12 | Visit the Natural History Museum |  |  |  |  |  |
| 13 | Visit the Aquarium |  |  |  |  |  |
| 14 | Read books about nature |  |  |  |  |  |
| 15 | Watch a nature documentary |  |  |  |  |  |
| 16 | Listen nature stories from the elders |  |  |  |  |  |
| 17 | Use phone or computer to inquire about animal and plant information |  |  |  |  |  |

亲爱的小朋友：

你好！

这是一份关于“人与自然关系”的调查问卷，非常开心你能够参与，请根据你的真实感受，在□中画“√”。 你只能从五个选项中选择一个答案，下面就开始答题吧！

**我的姓名**： **我的性别：**

**我的年龄**： **我的学校**：

|  | 【单选】根据你个人真实想法，用 “🗸” 选出你对下列陈述的看法 | 非常不同意 | 不同意 | 一般 | 同意 | 非常  同意 |
| --- | --- | --- | --- | --- | --- | --- |
| 1 | 我是自然世界的一部分 |  |  |  |  |  |
| 2 | 离开植物或动物，人类将无法生存 |  |  |  |  |  |
| 3 | 自然是动植物和人类共同的家园 |  |  |  |  |  |
| 4 | 当野生动物受到伤害时，我会感到伤心 |  |  |  |  |  |
| 5 | 我喜欢小动物们自由自在，而不是被关在笼子里 |  |  |  |  |  |
| 6 | 看到森林被砍伐，我会感到难过 |  |  |  |  |  |
| 7 | 身处户外会让我感到开心 |  |  |  |  |  |
| 8 | 我喜欢听大自然中的各种声音 |  |  |  |  |  |
| 9 | 我喜欢看大自然中的野花 |  |  |  |  |  |
| 10 | 当我感到难过时，我喜欢到户外去享受大自然 |  |  |  |  |  |
| 11 | 收集石头、贝壳等是很有趣的 |  |  |  |  |  |
| 12 | 我喜欢触摸动物和植物 |  |  |  |  |  |
| 13 | 照顾动物对我来说很重要 |  |  |  |  |  |

**【单选】在过去一年间，你多久做出一次以下行为？请在最接近的选项下打“🗸”**

|  |  | 从不 | 很少 | 有时 | | 经常 | 总是 |
| --- | --- | --- | --- | --- | --- | --- | --- |
| 1 | 我采取了一些行动去保护环境 |  |  |  |  | |  |
| 2 | 在我洗浴的时候，我尽量节约用水 |  |  |  |  | |  |
| 3 | 在学校时，我和老师同学们讨论采取行动来保护环境的重要性（如回收利用等） |  |  |  |  | |  |
| 4 | 在家中，我帮忙垃圾分类和回收利用 |  |  |  |  | |  |
| 5 | 当我不使用某些电器时，我关掉电源以节约能源 |  |  |  |  | |  |
| 6 | 我随手捡垃圾，去给自然生物一个更好的生活环境 |  |  |  |  | |  |
| 7 | 我曾把食物放在外面去喂鸟儿 |  |  |  |  | |  |
| 8 | 我在学校或者花园里给自然生物提供居巢（如虫子、刺猬等） |  |  |  |  | |  |
| 9 | 我将那些被困住的昆虫安全地放归野外 |  |  |  |  | |  |
| 10 | 我种植了一些昆虫和鸟儿可能喜欢的鲜花和植物 |  |  |  |  | |  |
| 11 | 我参与了一些保护鸟儿或昆虫的活动 |  |  |  |  | |  |
| 12 | 我是校内野生动物或自然小组的成员 |  |  |  |  | |  |
| 13 | 我是校外野生动物或自然小组的成员 |  |  |  |  | |  |

**【单选】在过去的一年间，你多久参加一次以下活动？ 请在最接近的选项下打“🗸”**

|  |  | 从不 | 很少 | 有时 | 经常 | 总是 |
| --- | --- | --- | --- | --- | --- | --- |
| 1 | 捉鱼虾、蝌蚪 |  |  |  |  |  |
| 2 | 爬树 |  |  |  |  |  |
| 3 | 摘野果品尝 |  |  |  |  |  |
| 4 | 玩泥巴 |  |  |  |  |  |
| 5 | 种植花草、树木 |  |  |  |  |  |
| 6 | 爬山 |  |  |  |  |  |
| 7 | 放风筝 |  |  |  |  |  |
| 8 | 收集自然物（贝壳，石头，叶子等） |  |  |  |  |  |
| 9 | 观察昆虫 |  |  |  |  |  |
| 10 | 参观动物园 |  |  |  |  |  |
| 11 | 参观植物园 |  |  |  |  |  |
| 12 | 参观自然博物馆 |  |  |  |  |  |
| 13 | 参观海洋馆 |  |  |  |  |  |
| 14 | 阅读自然相关的书籍 |  |  |  |  |  |
| 15 | 观看自然纪录片 |  |  |  |  |  |
| 16 | 听长辈讲自然的故事 |  |  |  |  |  |
| 17 | 用手机或电脑查询动植物的信息 |  |  |  |  |  |
