# Supplementary material for: Direct, indirect, and vicarious nature experiences collectively predict preadolescents’ self-reported nature connectedness and conservation behaviors
Source: PeerJ. 2023 Jun 21;11:e15542. doi: 10.7717/peerj.15542 (PMC10290449; doi:10.7717/peerj.15542)
Supplement: Supplemental Information 5 — Resident population density and local GDP per capital data gathered from the Statistics Bulletin of National Bureau of Statistics China 2020. Available at http://www.tjcn.org. [file peerj-11-15542-s005.docx]

**Table S1** Name of the schools investigated, type of residence, local per capita gross domestic product (GDP), resident population density, and sample size for girl/boy students in each school.

| No. | School name | Latitude/  Longitude | Sample size  (male/female) | City type | Per capita GDP  (RMB) ^*^ | Population density (person/km^2^) ^*^ |
| --- | --- | --- | --- | --- | --- | --- |
| 1 | Zhongxin, Menglun | 21°56′ N /101°14′ E | 153 (77/76) | Rural | 13672 | 42 |
| 2 | Changle, Menglun | 21°56′ N /101°14′ E | 127 (60/67) | Rural | 13672 | 42 |
| 3 | Second Primary School  Mengla | 21°29′ N /101°33′ E | 176 (89/87) | Rural | 41741 | 45 |
| 4 | 1^st^ Primary School Jinghong | 22°03′ N /100°47′ E | 112 (51/61) | City | 49553 | 94 |
| 5 | Shijijinyuan,Jinghong | 22°03′ N /100°47′ E | 163 (70/93) | City | 49553 | 94 |
| 6 | Zhugou, Xundian | 25°34′ N /103°11′ E | 163 (86/77) | Rural | 31223 | 128 |
| 7 | Erjie, Jinning | 24°41′ N /102°29′ E | 178 (103/75) | Rural | 56751 | 259 |
| 8 | Yunda, Kunming | 25°03′ N /102°42′ E | 153 (79/74) | City | 109473 | 2998 |
| 9 | Lianhua, Kunming | 25°32′ N /102°41′ E | 177 (91/86) | City | 109473 | 2998 |
| 10 | Wuhua, Kunming | 25°03′ N /102°40′ E | 189 (81/108) | City | 109473 | 2998 |
| 11 | Nanshan, Chunan | 29°35′ N /119°03′ E | 158 (83/75) | Rural | 52749 | 74 |
| 12 | Fenghuang, Hangzhou | 30°13′ N /120°10′ E | 145 (70/75) | City | 371597 | 13760 |
| 13 | Shengli, Hangzhou | 30°14′ N /120°12′ E | 155 (81/74) | City | 371597 | 13760 |
| 14 | Lijia, Jiande | 29°19′ N /119°02′ E | 77 (34/43) | Rural | 76853 | 191 |
| 15 | Cuiyuan, Hangzhou | 30°17′ N /120°07′ E | 49 (25/24) | City | 205128 | 3603 |

*Notes.* ^*^ Resident population density and local GDP per capital data gathered from the Statistics Bulletin of National Bureau of Statistics China 2020. Access from <http://www.tjcn.org>
