# Supplementary material for: Direct, indirect, and vicarious nature experiences collectively predict preadolescents’ self-reported nature connectedness and conservation behaviors
Source: PeerJ. 2023 Jun 21;11:e15542. doi: 10.7717/peerj.15542 (PMC10290449; doi:10.7717/peerj.15542)
Supplement: Supplemental Information 6 — DE, Direct nature experience; IDE, Indirect nature experience; VE, Vicarious nature experience; CC, Cognitive connection with nature; CE, Emotional connection with nature. PE, Pro-environmental behavior; PN, Pro-nature behavior. Extraction Method: Principal Component Analysis. Rotation Method: Varimax with Kaiser Normalization. Coefficients <0.300 suppressed. [file peerj-11-15542-s006.docx]

**Table S2 (a)** Reliability and validity tests of the *Nature Experience Scale*. Factor loadings and internal consistency alpha coefficients if items removed (*N* = 2,175).

| Factor loadings and internal consistency alpha if items removed coefficients | | | | |
| --- | --- | --- | --- | --- |
| Items | DE | IDE | VE | Alpha if items removed |
| Catch fish and tadpoles | 0.721 |  |  | 0.676 |
| Climb tree | 0.717 |  |  | 0.615 |
| Picking fruit to eat | 0.704 |  |  | 0.620 |
| Play mud | 0.718 |  |  | 0.617 |
| Planting flowers and trees | 0.721 |  |  | 0.676 |
| Mountaineer | 0.645 |  |  | 0.703 |
| Fly a kite | 0.688 |  |  | 0.687 |
| Collect natural things | 0.670 |  |  | 0.686 |
| Observe insects | 0.726 |  |  | 0.672 |
| Visit the Zoo |  | 0.846 |  | 0.863 |
| Visit the Botanical Garden |  | 0.834 |  | 0.861 |
| Visit the Natural History Museum |  | 0.842 |  | 0.857 |
| Visit the Aquarium |  | 0.813 |  | 0.875 |
| Read books about nature |  |  | 0.716 | 0.696 |
| Watch a nature documentary |  |  | 0.739 | 0.691 |
| Listen nature stories from the elders |  |  | 0.725 | 0.727 |
| Use phone or computer to inquire about animal and plant information |  |  | 0.744 | 0.714 |

*Notes*. * *DE* (*Direct nature experience); IDE (Indirect nature experience); VE (Vicarious nature experience).*

**Table S2 (b)** Reliability and validity tests of the modified version of the *Connection to Nature Index*. Factor loadings and internal consistency alpha coefficients if items removed (*N* = 2,175).

| Factor loadings and internal consistency alpha if items removed coefficients | | | |
| --- | --- | --- | --- |
| Items | CC | EC | Alpha if items removed |
| Humans are part of the natural world. | 0.518 |  | 0.669 |
| People cannot live without plants and animals. | 0.755 |  | 0.701 |
| Nature is the common home of animals, plants, and humans. | 0.598 |  | 0.677 |
| I feel sad when wild animals are hurt. | 0.509 |  | 0.648 |
| I like animals to be free, not caged. | 0.553 |  | 0.648 |
| It makes me sad to see deforestation. | 0.496 |  | 0.647 |
| Being outdoors makes me happy. |  | 0.566 | 0.728 |
| I like to hear different sounds in nature. |  | 0.726 | 0.709 |
| I like to see wild flowers in nature. |  | 0.756 | 0.701 |
| When I feel sad, I like to go outside and enjoy nature. |  | 0.685 | 0.714 |
| Collecting rocks and shells is fun. |  | 0.501 | 0.733 |
| I enjoy touching animals and plants. |  | 0.419 | 0.755 |
| Taking care of animals is important to me. |  | 0.499 | 0.728 |

*Notes*. * *CC* (*Cognitive connection with nature); CE (Emotional connection with nature).*

**Table S2 (c)** Reliability and validity test of the *Conservation Behaviors Scale.* Factor loadings and internal consistency alpha coefficients if items removed (*N* = 2,175).

| Factor loadings and internal consistency alpha if items removed coefficients | | | |
| --- | --- | --- | --- |
| Items | PE | PN | Alpha if items removed |
| I carry out activities to protect the environment. | 0.603 |  | 0.745 |
| To save water, I use less water when I take a shower or bath. | 0.721 |  | 0.750 |
| I talk to my teachers and peers at school about the importance of doing things to protect the environment. | 0.459 |  | 0.761 |
| I help separate (trash) and recycle at home. | 0.660 |  | 0.744 |
| I switch off electrical appliances when I am not using them to save energy. | 0.700 |  | 0.771 |
| I pick up litter to help nature have a better home. | 0.619 |  | 0.749 |
| I put food out to feed garden birds. |  | 0.433 | 0.827 |
| I make homes for nature at school or in the garden. |  | 0.606 | 0.815 |
| I put insects stuck inside safely outside. |  | 0.442 | 0.823 |
| I grow flowers and plants that birds and insects will like. |  | 0.496 | 0.820 |
| I take part in events to help nature (e.g. bird watching). |  | 0.614 | 0.811 |
| I am a member of a wildlife or nature group at school. |  | 0.842 | 0.820 |
| I am a member of a wildlife or nature group outside of school. |  | 0.850 | 0.813 |

*Notes*. * *PE* (*Pro-environmental behavior); PN (Pro-nature behavior).*

**Extraction Method: Principal Component Analysis. Rotation Method: Varimax with Kaiser Normalization. Coefficients < 0.300 suppressed.*
