# Supplementary material for: Direct, indirect, and vicarious nature experiences collectively predict preadolescents’ self-reported nature connectedness and conservation behaviors
Source: PeerJ. 2023 Jun 21;11:e15542. doi: 10.7717/peerj.15542 (PMC10290449; doi:10.7717/peerj.15542)
Supplement: Supplemental Information 7 — DE, Direct nature experience; IDE, Indirect nature experience; VE, Vicarious nature experience; CC, Cognitive connection with nature; CE, Emotional connection with nature; PE, Pro-environmental behavior; PN, Pro-nature behavior. Significance levels: <0.001***; <0.01**; <0.05*. [file peerj-11-15542-s007.docx]

**Table S3** Comparison of variables between girls and boys.

| Variables | Gender comparisons | | | | |
| --- | --- | --- | --- | --- | --- |
|  | Total sample  (*N* = 2,175)  Mean±SD | Girls  (*N* = 1,080)  Mean±SD | Boys  (*N* = 1,095)  Mean±SD | t value | p *value* |
| DE | 3.34±0.85 | 3.46±0.83 | 3.22±0.85 | 6.669 | *** |
| IDE | 3.24±1.17 | 3.30±1.16 | 3.18±1.18 | 2.325 | 0.020* |
| VE | 3.45±0.99 | 3.52±0.97 | 3.39±1.00 | 2.860 | 0.004** |
| CC | 4.52±0.51 | 4.56±0.47 | 4.47±0.55 | 4.404 | *** |
| EC | 4.25±0.62 | 4.37±0.55 | 4.14±0.66 | 8.537 | *** |
| PE | 3.82±0.73 | 3.86±0.71 | 3.78±0.74 | 2.462 | 0.014* |
| PN | 3.03±0.98 | 3.06±0.96 | 2.99±0.99 | 1.577 | 0.115 |

*Notes*. *DE* (*Direct nature experience); IDE (Indirect nature experience); VE (Vicarious nature experience); CC* (*Cognitive connection with nature); CE (Emotional connection with nature); PE (Pro-environmental behavior); PN (Pro-nature behavior).*

*Significance levels: < 0.001***; < 0.01**; <0.05*.*
