# Supplementary material for: Direct, indirect, and vicarious nature experiences collectively predict preadolescents’ self-reported nature connectedness and conservation behaviors
Source: PeerJ. 2023 Jun 21;11:e15542. doi: 10.7717/peerj.15542 (PMC10290449; doi:10.7717/peerj.15542)
Supplement: Supplemental Information 8 — DE, Direct nature experience; IDE, Indirect nature experience; VE, Vicarious nature experience; CC, Cognitive connection with nature; CE, Emotional connection with nature; PE, Pro-environmental behavior; PN, Pro-nature behavior. Significance levels: <0.001***; <0.01**; <0.05*. [file peerj-11-15542-s008.docx]

**Table S4** Comparison of variables across age.

| Variables |  | |  | | Age comparisons | | | | |
| --- | --- | --- | --- | --- | --- | --- | --- | --- | --- |
|  | Total sample  (*N* =2,175)  Mean±SD | Nine  (*N* =399)  Mean±SD | | Ten  (*N* =590)  Mean±SD | | Eleven  (*N* =615)  Mean±SD | Twelve  (*N* =571)  Mean±SD | *F* value | *p* value |
| DE | 3.34±0.85 | 3.44±0.84 | | 3.43±0.82 | | 3.30±0.84 | 3.21±0.89 | 8.594 | *** |
| IDE | 3.24±1.17 | 3.34±1.12 | | 3.29±1.14 | | 3.30±1.21 | 3.06±1.19 | 6.579 | *** |
| VE | 3.45±0.99 | 3.52±0.99 | | 3.41±0.96 | | 3.51±0.99 | 3.40±1.01 | 2.070 | 0.102 |
| CC | 4.52±0.51 | 4.52±0.51 | | 4.52±0.50 | | 4.52±0.52 | 4.50±0.53 | 0.348 | 0.791 |
| CE | 4.25±0.62 | 4.29±0.58 | | 4.28±0.57 | | 4.27±0.65 | 4.19±0.66 | 2.66 | 0.047* |
| PE | 3.82±0.73 | 3.85±0.70 | | 3.83±0.70 | | 3.87±0.74 | 3.73±0.76 | 4.221 | 0.006** |
| PN | 3.03±0.98 | 3.15±0.97 | | 3.04±0.96 | | 3.08±1.01 | 2.88±0.95 | 7.255 | *** |

*Notes*. *DE* (*Direct nature experience); IDE (Indirect nature experience); VE (Vicarious nature experience); CC* (*Cognitive connection with nature); CE (Emotional connection with nature); PE (Pro-environmental behavior); PN (Pro-nature behavior).*

*Significance levels: < 0.001***; < 0.01**; <0.05*.*
