# Supplementary material for: Direct, indirect, and vicarious nature experiences collectively predict preadolescents’ self-reported nature connectedness and conservation behaviors
Source: PeerJ. 2023 Jun 21;11:e15542. doi: 10.7717/peerj.15542 (PMC10290449; doi:10.7717/peerj.15542)
Supplement: Supplemental Information 9 — DE, Direct nature experience; IDE, Indirect nature experience; VE, Vicarious nature experience; CC, Cognitive connection with nature; CE, Emotional connection with nature; PE, Pro-environmental behavior; PN, Pro-nature behavior. Significance levels: <0.001***; <0.01**; <0.05*. [file peerj-11-15542-s009.docx]

**Table S5** Pearson correlations between variables (*N* = 2,175).

|  | DE | IDE | VE | CC | CE | PE | PN |
| --- | --- | --- | --- | --- | --- | --- | --- |
| DE | 1 |  |  |  |  |  |  |
| IDE | 0.632*** | 1 |  |  |  |  |  |
| VE | 0.630*** | 0.560*** | 1 |  |  |  |  |
| CC | 0.397*** | 0.302*** | 0.396*** | 1 |  |  |  |
| CE | 0.510*** | 0.377*** | 0.462*** | 0.632*** | 1 |  |  |
| PE | 0.552*** | 0.476*** | 0.605*** | 0.477*** | 0.489*** | 1 |  |
| PN | 0.586*** | 0.509*** | 0.601*** | 0.398*** | 0.493*** | 0.685*** | 1 |

*Notes. * DE* (*Direct nature experience); IDE (Indirect nature experience); VE (Vicarious nature experience); CC* (*Cognitive connection with nature); CE (Emotional connection with nature); PE (Pro-environmental behavior); PN (Pro-nature behavior);.*

*Significance* *levels: < 0.001***; < 0.01**; <0.05*.*
