# Supplementary figures and images for: Direct, indirect, and vicarious nature experiences collectively predict preadolescents’ self-reported nature connectedness and conservation behaviors
Source: PeerJ. 2023 Jun 21;11:e15542. doi: 10.7717/peerj.15542 (PMC10290449; doi:10.7717/peerj.15542)

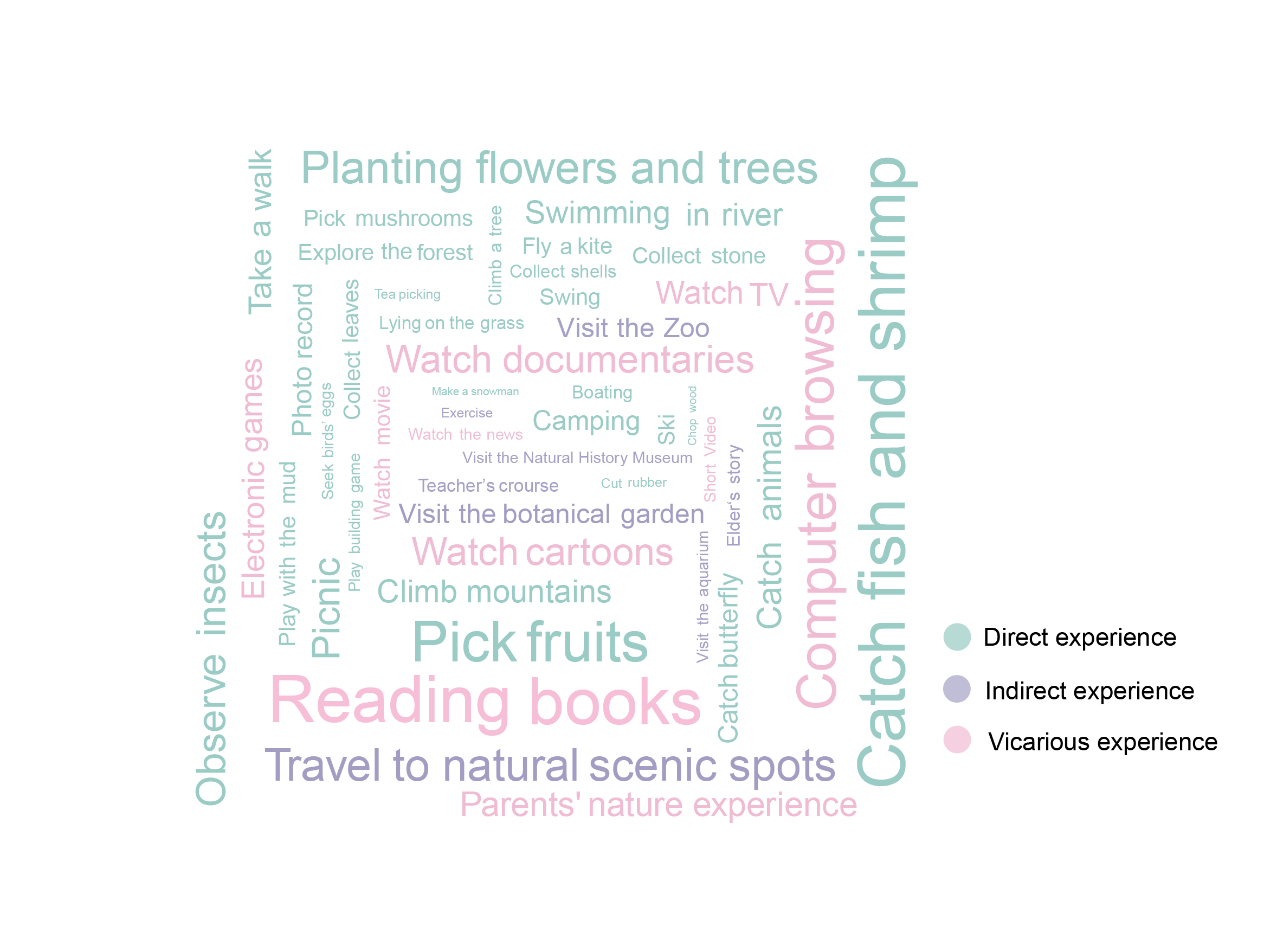

Supplement: Supplemental Information 10 — Extraction based on elements with frequency they mentioned. [file peerj-11-15542-s010.png]

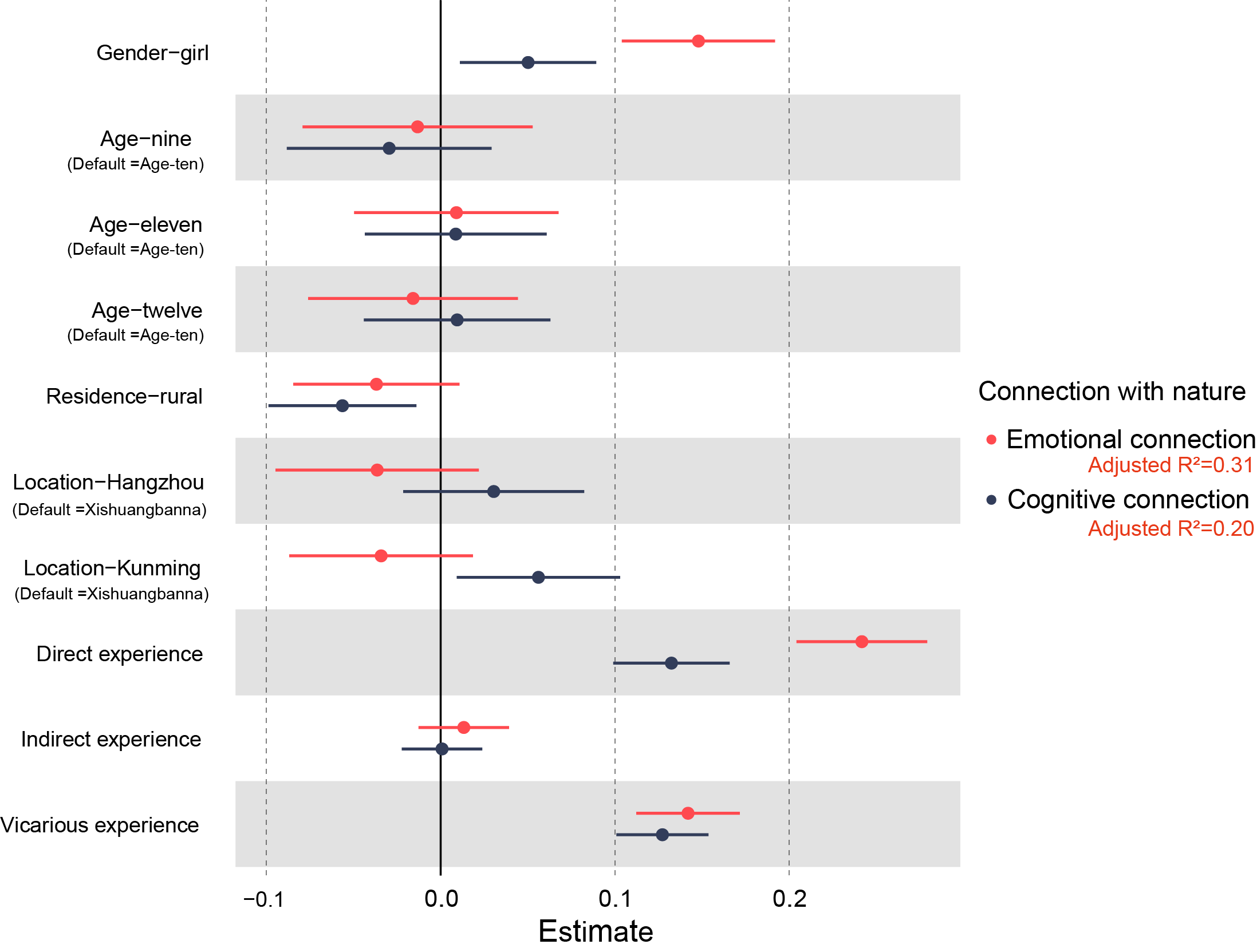

Supplement: Supplemental Information 11 [file peerj-11-15542-s011.png]
